# Unusual manifestations of young woman with MODY5 based on 17q12 recurrent deletion syndrome

**DOI:** 10.1186/s12902-022-00989-6

**Published:** 2022-03-26

**Authors:** Ying Cheng, Da-Peng Zhong, Li Ren, Hang Yang, Chen-Fu Tian

**Affiliations:** Department of Endocrinology, The General Hospital of Western Theater Command PLAJinniu DistrictSichuan Province, No. 270 Rongdu Avenue, Chengdu, 610083 People’s Republic of China

**Keywords:** Maturity-onset diabetes of the young 5 (MODY5), Hepatocyte nuclear factor 1 homeobox b gene (HNF1B), 17q12 Recurrent deletion syndrome

## Abstract

**Background:**

Maturity-onset diabetes of the young type 5 (MODY5) is a rare subtype of MODYs. It is caused by mutations of the hepatocyte nuclear factor 1 homeobox b gene (HNF1B). 17q12 recurrent deletion syndrome usually results in MODY5 because of the deletion of HNF1B. These patients often have other clinical manifestations besides diabetes. Refractory hypomagnesemia was a clue for further examination in this patient. But she lacked structural abnormalities of the genitourinary system and neurodevelopmental disorders that are common manifestations in patients with 17q12 recurrent deletion syndrome. Some atypical patients deserved attention.

**Case presentation:**

A 21-year-old young woman was admitted to our hospital for severe malnutrition and gastrointestinal symptoms. At age 20, she was diagnosed with type 2 diabetes mellitus (T2DM) and was administered oral antidiabetic drugs. Soon afterward, the patient discontinued the medication on her own accord and then went to the hospital again due to diabetic ketoacidosis. After insulin treatment, diabetic ketoacidosis was cured and blood glucose was controlled satisfactorily. But intractable nausea, vomiting, and persistent weight loss were stubborn. Further examination revealed that the patient had hypokalemia and hard rectification hypomagnesemia. Genetic testing revealed about 1.85 Mb heterozygous fragment deletion on chromosome 17 and deletion of exons 1–9 of HNF1B heterozygosity missing was approved. Finally, the patient was diagnosed MODY5.

**Discussion and Conclusions:**

The 17q12 recurrent deletion syndrome is characterized by MODY5, structural or functional abnormalities of the kidney and urinary tract, and neurodevelopmental or neuropsychiatric disorders. This patient did not have any structural abnormalities of the genitourinary system and neuropsychiatric disorders, which is rare. She had experienced a period of misdiagnosis before being diagnosed with 17q12 recurrent deletion syndrome, and hypomagnesemia was an important clue for her diagnosis. Therefore, diabetic physicians should be alert to a special type of diabetes if patients have unexplained signs and symptoms. The absence of well-known features of HNF1B disease does not exclude MODY5.

## Background

Maturity-onset diabetes of the young (MODY) is the most common type of monogenic diabetes that causes beta-cell dysfunction. Typically, the onset of MODYs is at an early age, with an average age of about 25. To the best of our knowledge, 14 candidate genes are involved in the pathogenesis of MODYs [1, 2]. Mutations in the hepatocyte nuclear transcription factor 1 homeobox α (HNF 1A), glucokinase gene (GCK) and hepato-nuclear factor 4 homeobox (HNF 4A) are the commonest subtype [3]. MODY5 is caused by mutation of the transcription factor HNF1B gene and is a rare subgroup of MODYs. It accounts for about 2–6% of patients with MODYs [4]. Nowadays, the name "MODY5" has often been replaced by HNF1B MODY. However, in keeping with some references, MODY5 was used in this paper. Heterozygous mutations and complete gene deletions in HNF1B each account for approximately 50% of all patients [4]. We herein discuss a case who suffered from HNF1B heterozygosity missing due to 17q12 recurrent deletion syndrome. HNF1B gene, located on chromosome 17, contains nine exons and encodes HNF1β, a member of the transcription factor superfamily containing homologous domains. HNF1β plays an important tissue-specific role in regulating gene expression in pancreas, kidney, intestine, liver and genital tract. It is also involved in embryonic development of these organs. The heterozygous mutation of HNF1B gene can cause abnormalities in the above organs. This patient with 17q12 recurrent deletion syndrome was diagnosed with MODY5, but lacked structural abnormalities of the genitourinary system, which is unusual. And she didn't have the neurodevelopmental disorders that are common in patients with 17q12 recurrent deletion syndrome. We shared the diagnosis and treatment process of this patient in order to provide some help to recognise some atypical patients in future clinical process.

## Case presentation

A 21-year-old young woman with diabetes was admitted to our hospital due to recurrent nausea and vomiting. She experienced polydipsia and polyuria a year ago and was diagnosed with diabetes mellitus. Initially she was treated with acarbose and blood glucose was not monitored. After six months, she discontinued antidiabetic drug at her own discretion. About two months ago, she was taken to the hospital in a coma and was diagnosed with diabetic ketoacidosis. Since then, insulin has been used to control blood glucose. Even though blood glucose control was under control, she still suffered from serious nausea, vomiting, abdominal pain and fatigue. Her body weight gradually lost 15 kg in two months. In order to improve these symptoms, the patient was admitted to our hospital. This patient’s maternal grandfather had been diagnosed with type 2 diabetes mellitus (T2DM), but her parents are in good health. There were no other family members with diabetes.

On admission, this patient was in a status of very cachectic nutritional. She was 161.0 cm tall and only 35.9 kg in weight. Body mass index (BMI) was only 13.85 kg/m^2^. Blood pressure was 111 vs. 54 mmHg. The patient has got serious malnutrition and muscular dystrophy. The physical examination of the heart and lungs showed no abnormalities. The patient complained of persistent lower abdominal pain, but the physical examination showed no abnormal abdominal signs.

After hospital admission, insulin was used consistently and blood glucose was well controlled. Both enteral and parenteral nutrition was given to improve nutritional status. A series of examinations were performed to seek the cause of nausea, vomiting, and weakness. At the same time, the etiology of the patient's diabetes was explored.

The patient's liver and kidney function were normal. Glycosylated hemoglobin (HbA1c) was 6.7% (normal reference value: 4.0 ~ 6.5%). Fasting serum insulin was 4.12 mU/L (normal reference value: 2.39 ~ 23.29 mU/L) and fasting serum c-peptide was 0.39 ng/ml (normal reference value: 0.81 ~ 3.85 ng/ml). Type 1 diabetes mellitus (T1DM) was excluded because of negative diabetes autoantibodies, including glutamate decarboxylase antibody (GADA), protein tyrosine phosphatase antibody (IA-2A), insulin antibody (IAA) and islet cell antibody (ICA). Hypomagnesemia and hypokalemia were found. Serum magnesium was 0.37 mmol/L (normal reference value: 0.7 ~ 1.1 mmol/L) and the fractional excretion of magnesium (FEMg) was 9.7% (normal reference value: < 4%). Serum potassium was 3.32 mmol/L (normal reference value: 3.5 ~ 5.3 mmol/L) and the fractional excretion of potassium (FEK) was 8.5% (normal reference value: < 10%). The patient's thyroid, parathyroid, pituitary, and adrenal functions were normal. Abdominal ultrasonography revealed intrahepatic calcification. Biliary system, pancreas, spleen and genitourinary system were normal. Gastroscopic examination revealed non-atrophic gastritis with bile reflux.

**Fig. 1:**
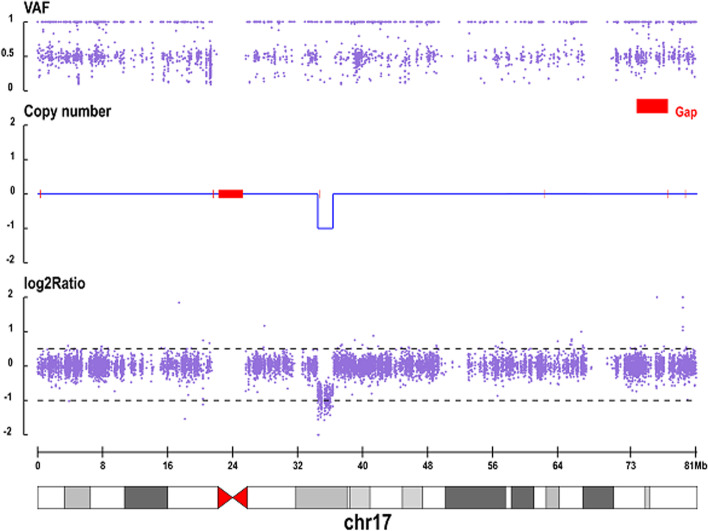
Microarray profile of chromosome 17 showing the deleted region. The whole exon region of the subject was sequenced by chip capture high-throughput sequencing method. The subject had a large fragment heterozygotic deletion on chromosome 17. The deletion interval was chr17: 34,493,374–36,347,081, about 1.85 Mb

Hypokalemia was quickly corrected after potassium chloride supplementation, but hypomagnesemia was refractory. Genetic testing was considered to screen for a specific type of diabetes due to the patient's refractory hypomagnesemia, unexplained abdominal pain and weight loss. After obtained informed consent from the patient, genetic testing was adopted. By the full exon detection method of chip capture high-throughput sequencing, there was a large heterozygous fragment deletion on chromosome 17, the deletion interval was chr17:34,493,374 to 36,347,081, about 1.85 Mb. And deletion of exons 1–9 of HNF1B heterozygosity missing was approved (Fig. 1).

Ultimately, MODY5 with HNF1B heterozygosity missing based on 17q12 recurrent deletion syndrome was diagnosed.

## Discussion

MODY is an autosomal dominant type of monogenic diabetes. It is caused by mutations in one of the genes that resulted in β-cell dysfunction [5]. The refined diagnostic criteria for MODY include: persistent hyperglycemia in early adulthood (typically before 30 years), clinical features incompatible with T1DM or T2DM, diabetes in at least one first-degree relative, evidence of residual pancreatic function, and absence of beta cell autoimmunity [1]. This patient met four criteria out of five standards. And none of her first-degree relatives were diagnosed with diabetes. But a second-degree relative was diagnosed with diabetes mellitus.

Although MODY is a most common monogenetic inherited diabetes, diagnosis is often delayed due to insidious onset of symptoms, lack of an obvious family history of diabetes and overlap of clinical features with T1DM and T2DM. Particularly accurate etiological diagnosis is challenging. Despite many biomarkers such as complement components, high-sensitivity C-reactive protein has been tested as auxiliary diagnostic tools, a definitive diagnosis depends on genetic testing [6]. This patient was initially diagnosed with type 2 diabetes and oral hypoglycemic medication was administered to control blood glucose. However, one year after the diagnosis of diabetes, diabetic ketoacidosis occurred and the function of pancreatic islet cell was failure. Although her maternal grandfather was diagnosed with T2DM, there were no first-degree relatives with diabetes. Therefore, MODY was not considered in the onset of disease, resulting in a delay in diagnosis. Therefore, it is necessary to screen out patients who need genetic testing to confirm the diagnosis of MODY, so that proper treatment can be given in time. Previous studies have shown that absence of islet autoantibodies (GAD insulinoma antigen-2, zinc transporter 8, and insulin autoantibodies), modest hyperglycemia and higher urinary c-peptide creatinine ratio, should result in genetic testing for MODY [7, 8]. This patient met at least two of these items.

Up to now, fourteen subtypes of MODY have been reported [5]. There is no concise or standardized diagnostic algorithm for MODY. However, some clinical signs indicate that the patient may have inherited diabetes. For example, there was hyperinsulinism during infancy, low triglyceride level, pancreatic agenesis, renal anomalies, genital anomalies and so on. This patient had serious fatigue, rapid weight loss, and refractory hypomagnesemia even after blood glucose was controlled. All these clinical characteristics suggest a specific type of diabetes. Genetic tests confirmed that the patient was MODY5 due to deletion of HNF1B gene.

Although MODY5 is inherited in an autosomal dominant pattern, genetic abnormalities can also occur spontaneously. It has been suggested that the new occurrence rate of HNF1B gene deletion can be as high as 50%. MODY5 is often one of the manifestations of 17q12 recurrent deletion syndrome. This patient is more likely to be spontaneous based on family history and final etiology.

17q12 recurrent deletion syndrome is also known as renal cysts and diabetes (RCAD) syndrome [9]. It is a distinct, recurrent chromosomal aberration. The aberration encompass multiple genes, HNF1B, LHX1, and ACACA, among others. Deletion of HNF1B is associated with MODY5. HNF1B is expressed in many organs. The spectrum of clinical phenotypes in patients with HNF1B mutations include abnormality of liver, kidney, pancreas and urogenital tract [10, 11]. There are other common features including delayed language development, learning disability, kidney involvement, and eye dysmorphism and strabismus [3]. The most prevalent findings in patients with 17q12 deletion were kidney abnormalities. It was found in about 80–85% patients of 17q12 recurrent deletion syndrome. This patient did not present common renal structural abnormalities, i.e. multicystic dysplastic kidney, solitary kidney, urinary tract malformation and so on, which was an important reason for the delayed diagnosis. However, this patient developed severe electrolyte disturbances, especially refractory hypomagnesemia, which is a characteristic manifestation of impaired renal tubular function [12]. HNF1B recognizes a site containing ion transport regulator 2 gene, which can cause hypomagnesaemia with hypermagnesuria and hypocalcinuria [13]. Motyka had reported four cases of MODY5 and these patients all had various degrees of hypomagnesaemia [14]. 74% of 17q12 recurrent deletion syndrome patients had renal insufficiency with hypomagnesaemia and/or hypocalcinuria [15]. Renal function tests and ultrasonography of the kidneys were required regularly in these patients. This patient suffered refractory hypomagnesaemia to further identify the cause and was proved to exist deletion of HNF1B gene. Hypomagnesemia is often the earliest and primary symptom of HNF1B-related disease. Renal structural abnormalities combined with hypomagnesemia are an important predictor for finding HNF1B mutations [16]. In addition, serum magnesium concentration is affected by age and sex. In girls, the serum magnesium concentration was generally higher than that of boys in the entire age spectrum. And hypomagnesemia is often underestimated in children patients [17]. The presence of hypomagnesemia is associated with renal tubular dysfunction, and hypokalemia, hyperuricemia, and proteinuria are also frequently obtained [18].

The HNF1B gene also plays an important role in the development of the pancreas. Many patients with 17q12 recurrent deletion syndrome have pancreatic atrophy and dysplasia. Pancreatic anomalies were difficult to detect by ultrasound, CT or magnetic resonance imaging should be performed. No pancreatic abnormalities were found on ultrasound in this patient and, it is a pity that abdominal CT/MRI was not performed. Exocrine pancreatic dysfunction is more common than pancreatic morphological abnormalities. When pancreatic exocrine dysfunction is present, patients often suffer from chronic abdominal pain, loose stools, and unintentional weight loss. The decreased faecal elastase-1 level was common in these patients, and gastrointestinal symptoms were recovered after trypsin supplementation. Low faecal elastase-1 concentration is a common feature of HNF1B gene defect even when MODY5 is not present [19]. This patient suffered from chronic abdominal pain and weight loss, exocrine pancreatic dysfunction was speculated. It is regrettable that faecal elastase-1 concentration was not measured in this patient.

There is no precise epidemiological data on 17q12 recurrent deletion syndrome. The prevalence of 17q12 recurrent deletion syndrome in Denmark was about 1.6 per 1,000,000 citizens [20]. The morbidity of 17q12 recurrent deletion syndrome in the Chinese population is unclear. But there are some case reports of 17q12 recurrent deletion syndrome. It was usually reported in fetuses with congenital renal abnormalities [21].

Neurological and psychiatric problems were common manifestations in patients with 17q12 deletion. About 40% of patients with 17q12 deletion syndrome present neurodevelopmental disorders [22]. It increases the risk of learning disability, mental retardation, autism, epilepsy and schizophrenia [23]. Other genes in the 17q12 region except HNF1B may be responsible for neurodevelopment and neuropsychiatric features. This patient showed normal intellectual development and no abnormal mental behavior. On account of absence of structural abnormalities of the genitourinary system and neurological manifestation, this patient was likely to be diagnosed with single MODY5 and follow-up of the kidney, pancreas, and nervous system is necessary.

Elevated liver enzymes were reported in 40% of individuals with 17q12 recurrent deletion syndrome [20, 24]. Live cysts, hepatomegaly, cholestasis, and steatohepatitis were also previously reported [20, 25]. This patient had no elevated liver enzymes and morphological abnormalities. Calcification plaque was found in her liver. Gastrointestinal diseases were less common features. Gastroesophageal reflux disease, duodenal atresia and hiatus hernia were reported previously [20, 24, 26]. Chronic non-atrophic gastritis with bile reflux was revealed under gastroscopy in this patient. About one third of females and a quarter of males with 17q12 recurrent deletion syndrome have genital abnormalities. However, genital abnormalities were not found in this patient.

Compared to the patients with point mutations in HNF1B, the patients of 17q12 recurrent deletion syndrome have earlier onset age, more symptoms and lower BMI [27]. 17q12 recurrent deletion syndrome may also co-exist with other genetic abnormalities. Cohen and colleagues have reported a monozygotic twin patient with Williams syndrome [28]. In addition to the typical deletion at 7q11.23, 1.7 Mb deletion in the 17q12 region was found on microarray comparative genomic hybridization [28].

The phenotype of 17q12 recurrent deletion syndrome is variable due to the differences of genotype [9]. The variation in severity of the disease makes the necessity for close surveillance of deletion carriers. It is regrettable that other relatives of this patient refused to accept relevant examinations to identify the presence of 17q12 deletion syndrome.

Due to the lack of specific clinical indicators for definitive diagnosis, genetic testing remains the gold standard for diagnosis. For these patients, genetic diagnosis can not only identify etiology, but also guide further screening for concomitant damage and modified treatment regimen. Insulin therapy and magnesium supplementation were essential for this patient. Because this disease is rare and genetic testing is expensive, the HNF1B score has been adopted to screen for suspicious patients. Seventeen indicators, including prenatal urogenital tract abnormalities, abnormalities of kidney or liver, electrolyte disorders and so on, were included to calculate HNF1B scores, helping to distinguish between mutated and nonmutated patients [12]. Raaijmakers and his colleagues came up with a simpler scoring system. Bilateral renal abnormality, renal cysts, simultaneous concomitant renal abnormalities and hypomagnesaemia were primary predictive for HNF1B mutations [16]. Based on these score criteria, this patient should be highly suspected of HNF1B-related disease. The HNF1B score is a simple tool to select patients for HNF1B gene analysis, but more research is needed to confirm the cutoff point to improve diagnostic accuracy [12, 29].

Whether 17q12 recurrent deletion syndrome existed or not, insulin is the main treatment for MODY5 on account of decreased islet function [30]. Although this patient was administered oral hypoglycemic drugs in the early stage, diabetic ketoacidosis manifested soon due to the severe deficiency of islet function. Therefore, insulin therapy was necessary.

## Conclusion

In summary, 17q12 recurrent deletion syndrome is a rare cause for diabetes mellitus type MODY5. It comprises variable combinations: MODY5, structural or functional abnormalities of the kidney and urinary tract, neurodevelopmental or neuropsychiatric disorders, and some unusual genomic syndrome. Although this patient lacked the typical manifestations of genitourinary tract impairment, diabetes with hypomagnesemia was an important clue to her diagnosis. Therefore, we need to pay attention to some atypical 17q12 recurrent deletion syndrome.

## Data Availability

The datasets used and/or analyzed during the current study are available from the corresponding author on reasonable request. The reported variation is based on the knowledge of human gene exon sequence and the theoretical system of variation classification at the time of reporting.

## Abbreviations

MODY: Maturity-onset diabetes of the young
HNF: Hepatocyte nuclear factor
T2DM: Type 2 diabetes mellitus
GCK: Glucokinase gene
BMI: Body mass index
HbA1c: Glycosylated hemoglobin
T1DM: Type 1 diabetes mellitus
GADA: Glutamate decarboxylase antibody
IA-2A: Protein tyrosine phosphatase antibody
IAA: Insulin antibody
ICA: Islet cell antibody
FEMg: Fractional excretion of magnesium
FEK: Fractional excretion of potassium
RCAD syndrome: Renal cysts and diabetes syndrome
